# Breakdown of the strong multiplet description of the Sm^2+^ ion in the topological Kondo insulator SmB_6_: specific heat studies

**DOI:** 10.1038/s41598-019-47776-3

**Published:** 2019-08-05

**Authors:** Ryszard J. Radwanski, Dawid M. Nalecz, Zofia Ropka

**Affiliations:** 1 Institute of Physics, Pedagogical University, Krakow, 30-084 Poland; 2 Center of Solid State Physics, Krakow, 31-150 Poland

**Keywords:** Electronic properties and materials, Topological insulators

## Abstract

We have theoretically confirmed the existence of in-gap real quantum-mechanical states in SmB_6_, which have been suggested by experiments. These in-gap states, below the hybridization gap of 20 meV, are related to the Sm^2+^ ion states and can be revealed by calculations within the spin-orbital |*LSL*_*z*_*S*_*z*_〉 space, with *L* = 3 and *S* = 3. Our approach overcomes difficulties related to the singlet *J* = 0 multiplet ground state. The in-gap states originate from the 49-fold degenerated term ^7^*F* (4*f *^6^), which is split by cubic crystal-field (CEF) and spin-orbit (s − o) interactions. There is competition between these interactions: the six-order CEF interactions produce a 7-fold degenerated ground state, whereas the s − o interactions, even the weakest one, produce a singlet (*J* = 0) ground state. We have found preliminary CEF and s − o parameters that produce the lowest states at 0 K (singlet) and 91 K (triplet) and the next triplet at 221 K, i.e., within the hybridization gap. The derived states well explain the large extra specific heat of SmB_6_, confirming the consistency and adequateness of our theoretical approach with the breakdown of the strong multiplet description of the Sm^2+^ ion in SmB_6_.

## Introduction

Studied for more than 50-years, the compound SmB_6_^1–4^ has recently become a strong candidate for a 3-dimensional topological Kondo insulator (TKI) with a robust bulk insulating gap^5–13^. The insulating gap in SmB_6_ is supposed to be created via the Kondo hybridization of localized Sm-4*f* and itinerant Sm-5*d* electrons, with the Fermi level residing in the hybridization gap. This situation is in contrast to that of a conventional band insulator^13^. The energy value of the hybridization gap depends on the experimental probe being approximately 20 meV (=232 K). However, some experiments point to the existence of in-(hybridization)gap states^14–17^ due to the observation of some lower-energy excitations. A 14-meV bulk collective mode observed within the hybridization gap by inelastic neutron scattering (INS) by Alekseev *et al*.^17^ has been recently interpreted as a spin exciton^18,19^.

Difficulty in understanding electronic and magnetic properties has led to a conclusion regarding SmB_6_ as a mixed-valence system in which the Sm ions rapidly fluctuate between non-magnetic Sm^2+^ (4*f *^6^) and magnetic Sm^3+^ (4*f *^5^ 5*d*^1^) electronic configurations, resulting in an average intermediate valence of 2.5–2.7. This valence can additionally change with temperature^20^. Recently, the temperature dependence of the specific heat *c*(T) of SmB_6_ was re-measured in two different laboratories on very good quality single crystals: in the USA^21^ and in Slovakia^22^.

In this contribution, we analyse the temperature dependence of the specific heat *c*(T) of SmB_6_ in a wide temperature range from 2 to 300 K, presenting, for the first time, a consistent explanation for the *c*(T) of SmB_6_ and the samarium contribution *c*_*Sm*_(T) to its specific heat.

## Results and Discussion

**Figure 1:**
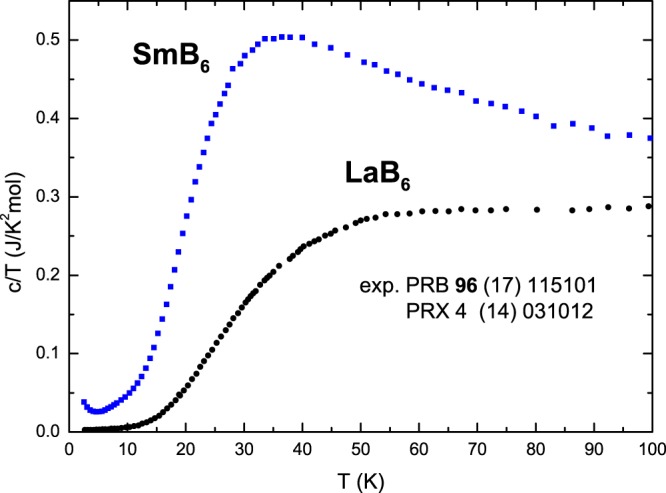
Experimental temperature dependence of the specific heat of SmB_6_ and LaB_6_, read from refs^21,22^, showing a very large excess specific heat of SmB_6_ compared to that of LaB_6_.

The experimentally derived specific heat of SmB_6_ exhibits a large extra heat compared with that of isostructural, nonmagnetic LaB_6_; see Fig. 1, which was redrawn from refs^21,22^. This excess heat in SmB_6_ is enormous because the entropy related to this excess, up to 300 K, amounts to 19–23 J/K mol f.u.^21^. It corresponds to 2.3–2.9 R (the gas constant R = 8.314 J/(K mol f.u.)). This extra entropy would point to a number of involved localized energy states from 10 to 16. These are large numbers totally not expected for the Sm^2+^ and Sm^3+^ ions. This problem was noticed 50 years ago^1–4^, and it is still under debate. In 2014, Phelan *et al*.^21^ wrote that “Some of the excess entropy will be due to the localization of the conduction electrons when the hybridization gap forms, but more likely explanation for the observed large value of Δ*S* is an additional phonon (lattice) contribution.” This additional phonon (lattice) contribution was not specified. In their recent 2017-year paper^22^, Orendac *et al*. mentioned only the existence of undefined in-(hybridization)gap states.

As has been restated by Sudermann *et al*.^23^, the CEF level scheme of the Sm^3+^ ion, which is a quantum system 4*f*^5^ with *L* = 3 and *S* = 5/2, in SmB_6_ is quite similar to that of the Ce^3+^ (4*f*^1^) ion, *L* = 3 and *S* = 1/2, in the isostructural CeB_6_^24^. In both cases, the spin-orbit interaction splits the 4*f* states into *J* = 5/2 and *J* = 7/2 multiplets. The *J* = 5/2 multiplet is further split by the cubic crystalline field into a Γ_7_ doublet and a ground-state Γ_8_ quartet. The energy difference between the Γ_8_ quartet and the excited Γ_7_ doublet is suggested to be approximately 15 meV for Sm^3+^ in SmB_6_ and 46 meV in CeB_6_, as shown in Fig. 1 of ref.^23^. The fine electronic structure for the Sm^2+^ (4*f*^6^) ion is even simpler. It is governed by the spin-orbit interactions, customarily assumed by a value of the spin-orbit coupling *λ*_*s*−*o*_ of 35 meV (=420 K), and due to the compensation of the orbital and spin momenta, the resultant ground multiplet is a singlet (*J* = 0). Considering that a *λ*_*s*−*o*_ value of 35 meV excited 6 multiplets, characterized by *J* values from 1 to 6, and with a (2*J* + 1) number of states, are at 35 meV (*J* = 1, triplet) and 105 meV (*J* = 2, quintuplet), other multiplets are at much higher energies.

It is obvious from this generally accepted description of the Sm^3+^ and Sm^2+^ ions that there is not so many CEF states below, say, 25 meV, as the experimental extra specific heat needs.

In our description, we also base our considerations on the CEF theory, thinking about the existence in SmB_6_ of samarium ions with an integer number of electrons of only 5 or 6, which are denoted as Sm^3+^ (4*f*^5^) and Sm^2+^(4*f*^6^) ions, respectively. However, we pay attention to the fact that the spin-orbit coupling in the case of the Sm^2+^ ion could be weaker than previously considered. The multiplet structure results from the very large spin-orbit coupling, a situation realized in most rare-earth compounds. Computer programs, in which the spin-orbit coupling can be given a finite value, are used in the description of 3*d* ions in, for instance, NiO and CoO oxides^25,26^, yielding the fine electronic structure and the orbital magnetic moment. This last outcome is important for describing the 3*d* magnetism. The Ni^2+^ and Co^2+^ ions are 3*d*^8^ and 3*d*^7^ quantum systems, respectively, and are both characterized by *L* = 3, similar to the 4*f*^6^ (Sm^2+^) quantum system.

In this contribution, we performed calculations of the fine electronic structure of the Sm^2+^ (4*f*^6^) ion in SmB_6_ within the spin-orbital |*LSL*_*z*_*S*_*z*_〉 space for the ^7^*F* term (*L* = 3 and *S* = 3) given by the two Hund’s rules. This fine electronic structure results from the combined action of the cubic crystal-field and the intra-atomic spin-orbit interactions and was calculated with the sole aim of describing the temperature dependence of the 4*f* contribution to the specific heat of SmB_6_.

The spin-orbital space of the Sm^2+^ ion is much bigger, i.e., 49 by 49, than the generally accepted ground state singlet multiplet *J* = 0 and the excited triplet multiplet *J* = 1 at 35 meV^23^.

**Figure 2:**
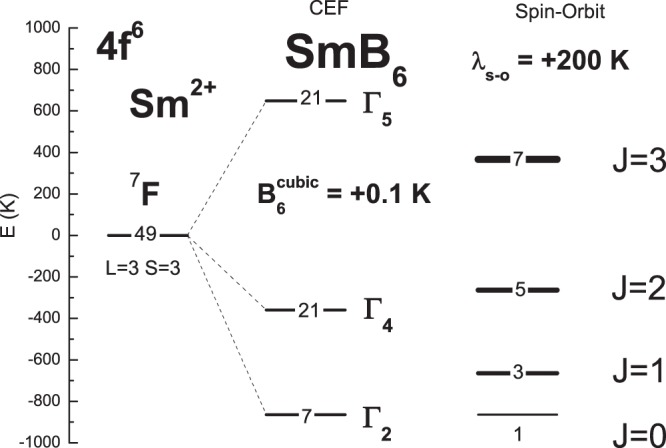
The energy states of the highly-correlated 4*f*^6^ electronic system associated with the 49-fold degenerated ^7^*F* term given by two Hund’s rules: *S* = 3 and *L* = 3, as the effect of the cubic crystal field with an exemplary six-order cubic CEF parameter *B*_6_ = +0.1 K (left) and as the sole effect of the spin-orbit coupling with an exemplary value of *λ*_*s−o*_ = +200 K (right). The spin-orbit multiplet structure has been shifted up by 1536 K to obtain the same energy as that of the lowest CEF level to show the involved possible excitation energies. The CEF Γ energies are −864 K, −360 K and +648 K. The multiplet structure is formed in the free ion, i.e., in the absence of CEF interactions.

The highly-correlated atomic-like 4*f*^+^ electronic system has the ^7^*F* ground term given by two Hund’s rules yielding *S* = 3 and *L* = 3. Its 49-fold degeneracy, 7 associated with the orbital degeneration times a 7-fold spin degeneracy, is lifted by intra-atomic spin-orbit interactions and, in a solid, by crystal-field interactions. The cubic crystal-field splits the 49 ^7^*F* states into 7 orbital states denoted as Γ_2_ (orbital singlet), Γ_4_ (orbital triplet) and Γ_5_ (orbital triplet)^27^. These Γ states have 7, 21, and 21 degeneracy, as shown in Fig. 2(left). On the other hand, the spin-orbit interactions split the 49 ^7^*F* states into 7 multiples denoted by *J* = 0, …, 6, as shown in Fig. 2(right). The lowest multiplet is a singlet *J* = 0, whereas excited multiplets are (2*J* + 1)-fold degenerated.

In Fig. 2, the six-order cubic CEF parameter, ${B}_{6}^{0}$ (and ${B}_{6}^{4}$ = −21 ${B}_{6}^{0}$ in the exact cubic symmetry), has been taken as +0.1 K, keeping the zero *B*_4_ term. A value of *λ*_*s*−*o*_ in Fig. 2 has been taken as +200 K, instead of the value of 420 K presented in the literature^23^, to show, for a better comparison, more multiplets resulting from the spin-orbit interactions.

From an inspection of Fig. 2, one sees that in the case of the Sm^2+^ ion, (a) the CEF and spin-orbit interactions are of comparable energies and thus must be treated on the same footing and, second, that (b) there is strong competition between them about the ground state and the number of states at the lowest energies.

**Figure 3:**
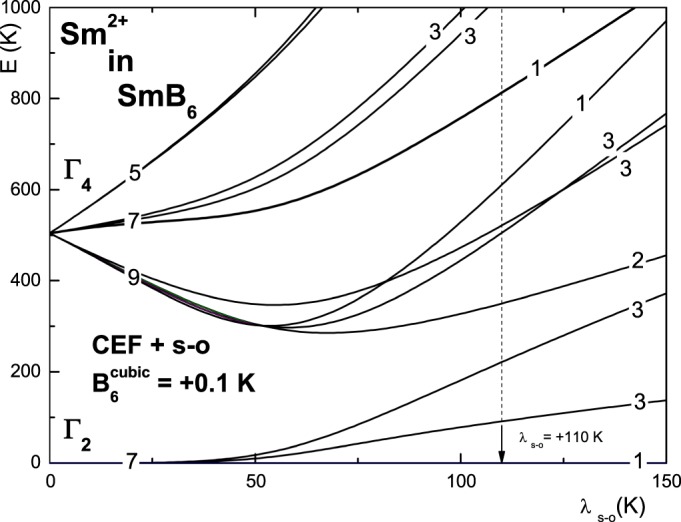
The calculated CEF +s−o energy states as a function of the spin-orbit coupling *λ*_*s*−*o*_ for six-order CEF interactions of the cubic symmetry *B*_6_ = +0.1 K with a zero *B*_4_ term. The change from the CEF electronic structure with the Γ_2_ ground state to the *J* = 0 and *J* = 1 multiplet structures is clearly visible. For *λ*_*s*−*o*_ = +110 K (10 meV), the lowest energy states are at 0 (singlet), 91 K (triplet), 221 K (triplet), 350 K (doublet), 505 K (triplet), 521 K (triplet), 610 K (singlet), ….. These energy states well reproduce the experimental temperature dependence of the samarium contribution to the specific heat of SmB_6_, as shown in Fig. 4.

The energy levels resulting from the combined action of the cubic CEF and spin-orbit interactions can be calculated by the standard single-ion CEF-like calculations^25,26,28–30^. The resulting fine electronic structure depends on three parameters: the fourth-order cubic CEF parameter *B*_4_, the six-order cubic CEF parameter *B*_6_ and the strength of the spin-orbit coupling *λ*_*s−o*_. In Fig. 3, we show the dependence of the fine electronic structure resulting from only the sixth-order *B*_6_ parameter, assuming that the fourth-order *B*_4_ parameter is zero, as a function of *λ*_*s*−*o*_. We have chosen the *B*_4_ parameter only, with a value of +0.1 K, from a didactic point of view to clearly show the idea of our approach and the resulting energy states. In Fig. 3, the change in the fine electronic structure from the CEF electronic structure with the Γ_2_ ground state to the *J* = 0 and *J* = 1 multiplet structure is clearly visible.

We have found preliminary values of *λ*_*s*−*o*_ = +110 K with cubic CEF ${B}_{6}^{0}$ = 0.1 K and ${B}_{6}^{4}$ = −2.1 K interactions (with *B*_4_ = 0) that well reproduce the experimental temperature dependence of the samarium contribution to the specific heat of SmB_6_. We obtained the lowest fine electronic structure with energy states at 0 K (singlet), 91 K (triplet), 221 K (triplet), 350 K (doublet), 505 K (triplet), 521 K (triplet), 610 K (singlet)….. It is clear that the 221 K triplet and the 350 K doublet will form the *J* = 2 multiplet for the large s − o coupling.

**Figure 4:**
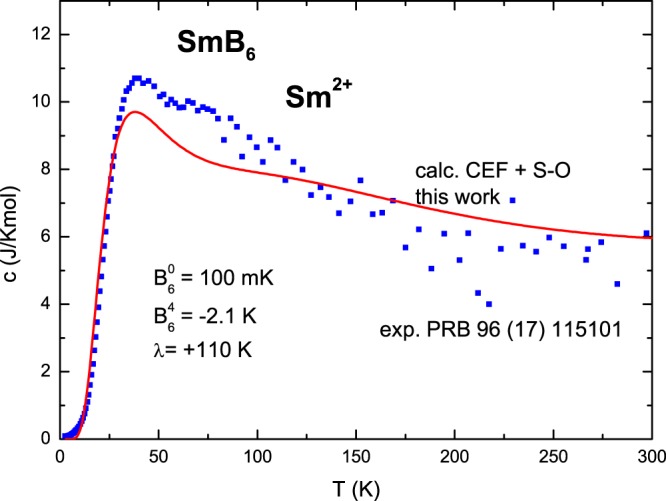
The calculated temperature dependence of the samarium 4*f* contribution to the specific heat of SmB_6_ associated with only Sm^2+^ ions. The combined action of exemplary cubic CEF (*B*_4_ = 0 K, *B*_6_ = +0.1 K) and s−o (*λ*_*s*−*o*_ = +110 K) interactions produce the lowest energy states at 0 (singlet), 91 K (triplet), 221 K (triplet), 350 K (doublet), 505 K (triplet), 521 K (triplet), 610 K (singlet), ….. Points are experimental results read from Fig. 1 in ref.^22^.

The temperature dependence of the resulting 4*f* contribution to the specific heat of SmB_6_ is shown in Fig. 4, together with experimental data read from Fig. 1 in ref.^22^. We conclude that our calculations, which can still be improved, very well describe the overall temperature dependence of the specific heat of SmB_6_. This indicates a quite surprising result: practically all Sm ions are in the divalent state. Leaving the problem of understanding of other than specific-heat phenomena for future studies, we conclude that our theoretical approach, with the spin-orbit coupling assumed to have a finite, relatively weak value, reveals a fine electronic structure with a quite large number of low-energy states.

## Conclusions

We have, for the first time, described the temperature dependence of the samarium 4*f* contribution to the specific heat of SmB_6_, explaining the large extra specific heat of SmB_6_ compared to that of LaB_6_, with a maximum at 40 K, which is in good agreement with experimental observations. We calculated the fine electronic structure of the Sm^2+^ (4*f*^6^) ion, finding 15 spin-orbital states within the lowest 50 meV, which are responsible for the large extra specific heat of SmB_6_. In our calculations, the finite, relatively weak strength of the relativistic spin-orbit coupling plays a fundamentally important role, indicating the breakdown of the strong multiplet description of the Sm^2+^ ion in SmB_6_. Our approach has theoretically confirmed the existence of the suggested in-(hybridization)gap states and given them physical meaning.
